# Effect of Match Location, Team Ranking, Match Status and Tactical Dimensions on the Offensive Performance in Spanish ‘La Liga’ Soccer Matches

**DOI:** 10.3389/fpsyg.2019.02089

**Published:** 2019-09-12

**Authors:** Joaquín González-Rodenas, Rodrigo Aranda-Malavés, Andres Tudela-Desantes, Ferran Calabuig Moreno, Claudio A. Casal, Rafael Aranda

**Affiliations:** ^1^Department of Recreation and Sport Pedagogy, Ohio University, Athens, OH, United States; ^2^Department of Physical Education and Sports, University of Valencia, Valencia, Spain; ^3^Doctoral School, Valencia Catholic University “Saint Vincent Martyr”, Valencia, Spain; ^4^Department of Science of Physical Activity and Sport, Catholic University of Valencia, San Vicente Mártir, Valencia, Spain

**Keywords:** soccer, scoring opportunitie, goal, game strategy, match analysis, observational methods

## Abstract

The aim of this paper was to study the combined effects of tactical and contextual dimensions on achieving offensive performance in open play possessions from Spanish “La Liga” soccer matches. 1860 team possessions from 20 random matches were evaluated by means of multidimensional observation. Multilevel regression models were constructed to predict the probability to achieve offensive performance according to the tactical and contextual dimensions registered in each possession. Performing penetrative actions after recovering the ball (OR = 1.497; 95% CI: 1.022–2.192; *P* < 0.05), and progressing by fast attacks (OR = 3.588; 95% CI: 2.045–6.294; *p* < 0.001) or counterattacks (OR = 7.097; 95% CI: 3.530–14.269; *P* < 0.001) was more effective to create scoring opportunities than performing a non-penetrative action and progressing by combinative attack, respectively. Also, progressing by long possessions (OR = 5.057; 95% CI: 2.406–10.627; *p* < 0.001) was more effective than progressing by short possessions to create scoring opportunities. As for contextual dimensions, multivariate analyses showed how playing at home and against high-ranked opponents registered more likelihood of achieving offensive penetration, although no associations were found in the production of scoring opportunities. Tactical dimensions as initial penetration, type of attack and possession length played an important role on achieving offensive penetration and goal scoring opportunities in Spanish Soccer “La Liga”.

## Introduction

The style of play in soccer is the characteristic playing pattern demonstrated by a team during games and it shows players and ball movements, interaction of players, as well as elements of speed, time and space (Hewitt et al., 2016). Although each style of play is relatively stable during different games or tactical situations, it has been shown how contextual variables as venue, quality of opposition and match status influence the use of styles of play in soccer match play (Fernandez-Navarro et al., 2018). Moreover, the interaction with the opposing team tactics creates a specific and complex context that may modulate and influence the teams’ style of play during the game.

The analysis of playing styles is arising in recent years (Gómez et al., 2018; Yang et al., 2018), but there are still very few studies that describe the effectiveness of playing tactics to produce offensive performance in professional soccer. The existing literature based on observational methodology, revealed that counterattacks (Tenga et al., 2010; González-Rodenas, 2013) and fast attacks (Sarmento et al., 2018) were more effective to create offensive performance than positional attacks in the Norwegian, American and top European teams, respectively.

Nevertheless, the different cultural, historical and social factors of each country make different the way of understanding soccer and implementing styles of play in each regional competition (Sarmento et al., 2013). In this sense, Spanish La Liga (SLL) seems to have a more “possession-based” style of play in comparison to English Premier League or Italian Serie A (Sarmento et al., 2013; Mitrotasios et al., 2019). In the last decade, SLL has achieved to occupy the highest position in the indices related with international prestige and the competitive quality of teams, followed by German Bundesliga, English Premier League and Italian Serie A (Vales-Vázquez et al., 2017). However, according to our knowledge, no study up to date has specifically analyzed the effectiveness of playing tactics to achieve offensive performance in this competition. Furthermore, contextual variables such as match location, match status and quality of opposition have been shown to influence the tactical performance and success of teams during competition (Fernandez-Navarro et al., 2018). Despite this, very few studies have evaluated how the contextual factors can affect the offensive effectiveness.

In this vein, further research is needed to analyse the effectiveness of playing tactics and contextual variables in different countries and professional competitions. For that purpose, numerous studies have shown that systematic observation is an adequate methodology for analyzing tactical behaviors in sport (Anguera and Hernández-Mendo, 2013) because permits the inclusion of categorical data from the qualitative evaluation of different dimensions of match performance, and may improve our ability to describe soccer match play actions (Grehaigne et al., 2001; Hughes and Bartlett, 2002; Suzuki and Nishijima, 2004; Sarmento et al., 2014). For the analysis of data, the study of the combined effects of offensive, defensive and contextual variables allow to create statistical models that capture the interdependency and interaction between different dimensions to achieve offensive outcomes.

Therefore, the aim of this paper was to study the combined effects of match location, team ranking, match status and tactical dimensions on achieving offensive performance in open play possessions from Spanish Soccer “La Liga” matches by using multidimensional qualitative analysis. It is hypthesized that high-ranked teams and playing at home present higher offensive effectinesses. Regarding the effect of tactical dimensions, the hypothesis is that team sequences that start with initial penetration and progress by counterattack have higher odds of creating scoring opportunities.

## Materials and Methods

### Sample

The context of the analysis was the Spanish “La Liga” 2017–2018. This competition has 20 teams that play a total of 380 matches. The unit of analysis was a “team possession” that started in an open play situation according to the definition of Pollard and Reep (1997, p. 542).

“A team possession starts when a player gains possession of the ball by any means other than from a player of the same team. The player must have enough control over the ball to be able to have a deliberate influence on its subsequent direction. The team possession may continue with a series of passes between players of the same team but ends immediately when one of the following events occurs: (a) the ball goes out of play; (b) the ball touches a player of the opposing team (e.g., by means of a tackle, an intercepted pass or a shot being saved). A momentary touch that does not significantly change the direction of the ball is excluded”.

In this way, each match from the Spanish “La Liga” was assigned with a number from 1 to 380. An online random number generator (Research Randomizer 4.0; Urbaniak and Plous, 2013) was used to select 20 random matches. The selected matches were downloaded from Wyscout platform (Wyscout Spa, Italy). A total of 3520 team possessions were evaluated After excluding those possessions that started by means of restarts and set pieces, 1860 team possessions (52.8%) that started in open play were included in the study.

### Variables

Four independent tactical dimensions (Table 1) were evaluated based on the observational tool REOFUT (Aranda et al., 2019). These variables are related to the offensive behavior during the start (initial penetration and initial opponent pressure) and the development of the team possession (type of attack and duration of the attack). Additionally, the effect of three independent contextual dimensions was analyzed (match location: “*home; away*,” situational match status during the match: “*losing, drawing, winning*” and quality of opponent “*high-ranked*: from first position to fifth position in the final standing; *medium-ranked*: from sixth position to fifteenth position; *Low-ranked*: from sixteenth position to twentieth position”).

**TABLE 1:** Descriptions and definitions of tactical dimensions and categories (independent variables).

**Initial Penetration:** degree of offensive depth in the first three seconds of the team possession: 1. **Penetrative action:** Passes or dribbles toward the opponent’s goal past opponent player(s) performed during the first three seconds of the ball possession. 2. **Non-penetrative action:** any technical action toward any direction that does not past opponent player(s) performed during the first three seconds of the ball possession.
**Initial Opponent pressure: d**istance between the player/s with the ball (first attackers) and the immediate pressing opponent player(s) (first defender(s)) during the first three seconds of the ball possession. 1. **Initial pressure:** one or several opponent players pressure the attackers within the first 3 s of the possession (the defender(s) are always located within 1.5 m of the first attackers). 2. **Non-initial pressure:** any player pressures the attacker (s) during the first 3 s of the possession.
**Duration of the attack: d**uration of the offensive sequence in seconds. Four categories were considered: 1. **Very short** (0–10 s), 2. **Short** (11–20 s): 3. **Long** (21–30 s) and 4. **Very long** (31 or more seconds)
**Type of attack: d**egree of offensive directness (Tenga et al., 2010; Lago-Ballesteros et al., 2012; González-Ródenas et al., 2015; Sarmento et al., 2018) in the offensive process. Three categories were considered: 1. **Combinative attack:** (a) the progression toward the goal has a high percentage of non-penetrative and short passes, (b) the circulation of the ball takes place more in width than in depth (Sarmento et al., 2018), (c) the intention of the team is to disorder the opponent using a high number of passes and slow tempo (evaluated qualitatively) and (d) the opposing team has the opportunity to minimize surprise, reorganize his system and be prepared defensively 2. **Direct attack: (**a) the progression toward the goal is based on one long pass from the defensive players to the forward players (evaluated qualitatively), (b) the circulation of the ball takes place more in depth than in width, (c) the intention of the team is to take the ball directly near the goal area to have opportunities of finishing by using a reduced number of passes and high tempo and (d) the opposing team has the opportunity to minimize surprise, reorganize his system and be prepared defensively. 3. **Fast attack:** (a) the progression toward the goal has a high number of penetrative and short passes, (b) the circulation of the ball takes place in width and depth (Sarmento et al., 2018), (c) the intention of the team is to disorder the opponent with a reduced number of passes and high tempo (evaluated qualitatively) and (d), the opposing team has the opportunity to minimize surprise, reorganize his system and be prepared defensively. 4. **Counterattack:** (a) the progression toward the goal attempts to utilize a degree of imbalance right from start to the end with high tempo (Tenga et al., 2010), (b) the circulation of the ball takes place more in depth than in width, (c) the intention of the team is to exploit the space left by the opponent when they were attacking, and (d) the opposing team does not have the opportunity to minimize surprise, reorganize his system and be prepared defensively.


**FIGURE 1:**
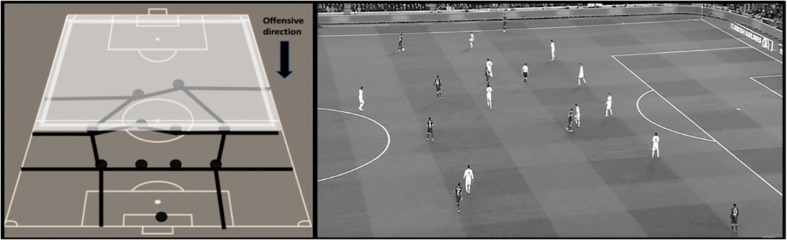
Graphic representation and real example of no offensive penetration. The team possession does not achieve to disorder and beat the forward or midfielders’ lines of the opposing team during the offensive sequence.

**FIGURE 2:**
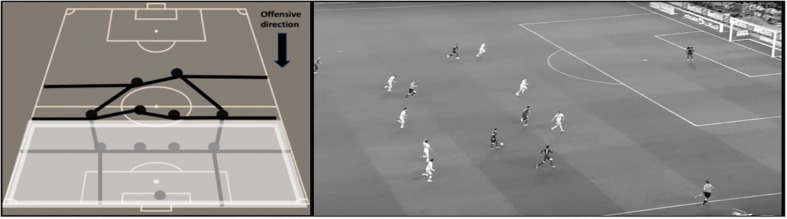
Graphic representation and real example of offensive penetration. The team possession achieves to beat the forward and midfielders’ lines of the opponent and face directly the defensive line during the offensive sequence but the possession ends without creating any scoring opportunity. The player(s) facing the defensive line has/have enough time and space to perform intended actions on the ball at the moment of receiving the ball.

**FIGURE 3:**
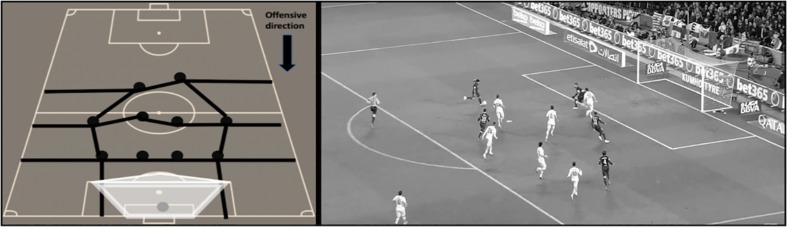
Graphic representation and real example of scoring opportunity. The team has a clear chance of scoring a goal during the ball possession. This includes all goals, all shots produced inside the score pentagon^∗^, those shots produced outside the score pentagon that pass near the goal (evaluated qualitatively) and all chances of shooting inside the score pentagon (the player is facing the goal, there are not any opponents between him and the goal and he has enough space and time to make a playing decision). ^∗^ Score pentagon is used as the zone of reference because it selects the space with high shooting angle and a short distance to goal (20 meters or less) which are very important factors to achieve goals (Pollard et al., 2004; Ensum et al., 2005).

For the possession outcome, the dependent variable “offensive performance” was evaluated. This variable analyzes the degree of penetration over the opposing defense as well as the creation of goal scoring opportunities during the team possession. This variable comprised three categories: (1) No offensive penetration (Figure 1), (2) Offensive penetration (Figure 2), and (3) Scoring opportunity (Figure 3).

### Match Performance Analysis

The study is based on observational methodology (Anguera and Hernández-Mendo, 2013; Anguera et al., 2017). The software Lince (Gabin et al., 2012) was used to observe, code and register the data during the evaluation process. For the analysis, a researcher with experience in match analysis and soccer coaching completed a theoretical and practical training on the use of the REOFUT instrument. Inter-observer and intra-observer (observers analyzed the sample again after one month) analyses showed appropriate levels of reliability for the tactical variables analyzed in the study based on Cohen’s Kappa calculations after the analysis of one complete match (Celta-Villareal CF) (initial penetration: 0.819, 0.963; initial defensive pressure: 0.815, 0.816; duration of the attack: 0.958, 0.963; type of attack: 0.776, 0.898, for inter and intra-observer reliability, respectively).

### Statistical Analysis

All the analyses were performed using SPSS software (IBM SPSS, Version 20.0). An analysis of frequencies was carried out to describe the characteristics of the sample and the occurrence of each tactical dimension according to the offensive performance.

Multilevel modeling was carried out to cluster the ball possessions (Level 2) within teams (Level 1). Firstly, multinomial logistic regressions were constructed to predict three different levels of offensive penetration (0. no offensive penetration, 1. offensive penetration and 2. scoring opportunity). Secondly, binary logistic regressions were performed to predict the possibility of achieving scoring opportunities (0 = no scoring opportunity, 1 = scoring opportunity). Unadjusted models (univariate analysis) were carried out to determine the association of each independent variable with the dependent variable. Based on the unadjusted models, adjusted logistic multilevel models (multivariate analysis) were constructed with all significant independent variables from the unadjusted models included.

## Results

Table 2 shows the descriptive analysis of the sample. Greater number of team possessions faced initial opponent pressure, the majority of initial actions were not penetrative and the possessions length was predominantly very short (10 or less seconds) or short (11–20 s). Regarding the type of attack, combinative and fast attacks comprised nearly 75% of the team possessions. In this context, 50.4% of the team possessions achieved to penetrate offensively, while 10.4% created a goal scoring opportunity.

**TABLE 2:** Descriptive characteristics of the sample.

**Dimension**	**N**	**No scoring opportunity**		**Scoring opportunity**
		**No offensive penetration**	**Offensive penetration**	
		**N (%)**	**N (%)**	**N (%)**
**Initial penetration**				
No penetration	1034 (55.6)	471 (45.6)	491 (47.5)	72 (7.0)
Penetration	826 (44.4)	258 (31.2)	447 (54.1)	121 (14.6)
**Initial opponent pressure**				
Initial Pressure	1417 (76.2)	630 (44.5)	651 (45.9)	136 (9.6)
Non-initial pressure	443 (23.8)	99 (22.3)	287 (64.8)	57 (12.9)
**Duration of the attack**				
0–10 s	907 (48.8)	491 (54.1)	336 (37.0)	80 (8.8)
11–20 s	514 (27.6)	139 (27.0)	310 (60.3)	65 (12.6)
21–30 s	264 (14.2)	65 (24.6)	173 (65.5))	26 (9.8)
31 + s	175 (9.4)	34 (19.4)	119 (68.0)	22 (12.6)
**Type of attack**				
Combinative attack	661 (35.5)	243 (36.8)	363 (54.9)	55 (8.3)
Direct attack	354 (19.0)	213 (60.2)	135 (38.1)	6 (1.7)
Counterattack	205 (11.0)	66 (32.2)	95 (46.3)	44 (21.5)
Fast attack	604 (34.4)	207 (32.3)	345 (53.9)	88 (13.8)
**Match location**				
Away	919 (49.4)	389 (42.3)	437 (47.6)	93 (10.1)
Home	941 (50.6)	340 (36.1)	501 (53.2)	100 (10.6)
**Quality of opposition**				
Low-ranked	500 (26.9)	201 (40.2)	241 (48.2)	58 (11.6)
Medium-ranked	885 (47.6)	360 (40.7)	439 (49.6)	86 (9.7)
High-ranked	475 (25.5)	168 (35.4)	258 (54.3)	49 (10.3)
**Match status**				
Losing	358 (19.2)	122 (34.1)	203 (56.7)	33 (9.2)
Drawing	1110 (59.7)	437 (39.4)	563 (43.9)	110 (9.9)
Winning	392 (21.1)	170 (43.4)	172 (43.9)	50 (12.8)
**Total**	1860	729 (39.2)	938 (50.4)	193 (10.4)

Table 3 shows the explanatory variables that predict offensive penetration in comparison with the team possessions that did not achieve offensive penetration. Multivariate analysis showed that performing initial penetration, playing against no opposing pressure, sustaining long possessions and attacking with fast attacks or counterattacks was more effective to achieve offensive penetration than non-performing initial penetration, playing against opposing pressure, building short possessions or attacking by means of combinative attacks, respectively. For contextual dimensions, playing at home and against a high-ranked team registered higher probabilities in achieving offensive penetration, in comparison with playing away and playing versus low-ranked teams, respectively.

**TABLE 3:** Multilevel multinomial logistic regression predicting to achieve offensive penetration vs no penetration (Reference category).

**Dimension**	**Offensive penetration vs. penetration (univariate analysis)**			**Offensive penetration vs. penetration (multivariate analysis)**		
	**β**	**SE**	**OR (95% CI)**	**β**	**SE**	**OR (95% CI)**
**Initial penetration**						
No penetration (Ref)						
Penetration	0.526	0.104	1.690 (1.378–2.073)^∗∗∗^	0.429	0.145	1.536 (1.155–2.041)^∗∗^
**Initial pressure**						
Initial Pressure (Ref)						
Non-initial pressure	1.036	0.141	2.818 (2.139–3.713)^∗∗∗^	1.044	0.164	2.839 (2.056–3.920)^∗∗∗^
**Duration of the attack**						
0–10 s (Ref)						
11–20 s	1.181	0.128	3.259 (2.538–4.180)^∗∗∗^	1.375	0.162	3.954 (2.878–5.433)^∗∗∗^
21–30 s	1.495	0.168	4.463 (3.213–6.200)^∗∗∗^	2.098	0.229	8.148 (5.19612.777)^∗∗∗^
31 + s	1.836	0.213	6.240 (4.106–9.481)^∗∗∗^	2.454	0.276	11.639 (6.769–20.215)^∗∗∗^
**Type of attack**						
Combinative (Ref)						
Direct attack	–1.189	0.152	0.305 (0.226−0.411)^∗∗∗^	–0.065	0.198	0.937 (0.635−1.381)
Fast attack	–0.066	0.133	0.935 (0.721−1.213)	1.049	0.195	2.854 (1.945−4.187)^∗∗∗^
Counterattack	0.087	0.208	1.089 (0.724−1.638)	1.480	0.284	4.395 (2.519−7.669)^∗∗∗^
**Match location**						
Away (Ref)						
Home	0.412	0.122	1.487 (1.170–1.891)^∗∗^	0.387	0.165	1.472 (1.065–2.035)^∗^
**Quality of opposition**						
Low-ranked (Ref)						
Medium-ranked	0.057	0.143	1.059 (0.799–1.403)	0.136	0.178	1.145 (0.808–1.623)
High-ranked	0.507	0.169	1.660 (1.191–2.314)^∗∗^	0.424	0.198	1.527 (1.035–2.254)^∗^
**Match status**						
Losing (Ref)						
Drawing	–0.246	0.150	0.783 (0.583–1.051)	–0.108	0.179	0.897 (0.631−1.275)
Winning	–0.405	0.184	0.663 (0.462–0.952)^∗^	–0.349	0.229	0.706 (0.451−1.105)
**Intercept**				–1.597	0.315	0.203 (0.109–0.376)^∗∗∗^

*β, Coefficient; SE, Standard error; OR, Odds Ratio; CI, Confidence interval for odds ratio; ^∗^*p* < 0.05, ^∗∗^*p* < 0.01, ^∗∗∗^*P* < 0.001.*

Table 4 shows the explanatory dimensions that predict the creation of scoring opportunities in comparison with those possessions that did not achieve offensive penetration. The multivariate analysis indicated that tactical dimensions as performing initial penetration, attacking without initial opponent pressure, sustaining longer team possessions and advancing by means of fast attacks or counterattacks obtained more likelihood of creating goal scoring opportunities than their counterparts. No differences were observed in the odds ratio of producing scoring opportunities for the contextual dimensions.

**TABLE 4:** Multilevel multinomial logistic regression predicting to achieve scoring opportunity vs. no penetration (Reference category).

**Dimension**	**Scoring opportunity vs. no penetration (univariate analysis)**			**Scoring opportunity vs. no penetration. (multivariate analysis)**		
	**β**	**SE**	**OR (95% CI)**	**β**	**SE**	**OR(95% CI)**
**Initial penetration**						
No penetration (Ref)						
Penetration	1.154	0.173	3.172 (2.260–4.452)^∗∗^	0.630	0.228	1.877 (1.200–2.937)^∗∗^
**Initial pressure**						
Initial Pressure (Ref)						
Non-initial pressure	1.020	0.208	2.772 (1.844–4.167)^∗∗^	1.168	0.242	3.217 (2.002–5.169)^∗∗∗^
**Duration of the attack**						
0–10 s (Ref)						
11–20 s	1.047	0.196	2.848 (1.940–4.182)^∗∗∗^	1.679	0.251	5.359 (3.273–8.774)^∗∗∗^
21–30 s	0.780	0.279	2.182 (1.263–3.769)^∗^	2.345	0.388	10.430 (4.873–22.326)^∗∗∗^
31 + s	1.437	0.308	4.209 (2.302–7.696)^∗∗^	3.089	0.451	21.953 (9.060–53.191)^∗∗∗^
**Type of attack**						
Combinative (Ref)						
Direct attack	–2.065	0.449	0.127 (0.053–0.306)^∗∗∗^	–0.741	0.486	0.477 (0.184–1.237)
Fast attack	0.680	0.214	1.973 (1.297–3.003)^∗∗^	1.953	0.327	7.049 (3.712–13.387)^∗∗∗^
Counterattack	1.293	0.277	3.645 (2.118–6.274)^∗∗∗^	2.889	0.420	17.981 (7.884–41.010)^∗^
**Match location**						
Away (Ref)						
Home	0.502	0.189	1.652 (1.139–2.395)^∗∗^	0.137	0.241	1.146 (0.715–1.839)
**Quality of opposition**						
Low-ranked (Ref)						
Medium-ranked	–1.131	0.219	0.877 (0.571–1.348)			
High-ranked	0.341	0.254	1.406 (0.855–2.313)			
**Match status**						
Losing (Ref)						
Drawing	–0.202	0.240	0.817 (0.511-1.308)			
Winning	0.041	0.277	1.042 (0.605-1.792)			
**Intercept**				–4.062	0.492	0.017 (0.007-0.045)^∗∗∗^

*β, Coefficient; SE, Standard error; OR, Odds Ratio; CI, Confidence interval for odds ratio; ^∗^*p* < 0.05, ^∗∗^*p* < 0.01, ^∗∗∗^*P* < 0.001.*

Table 5 shows the explanatory variables that predict the creation of scoring opportunities in comparison with those possessions that achieved offensive penetration. Tactical dimensions as sustaining long possessions (31 s or more) and advancing by means of fast attack or counterattack were more likely to produce scoring opportunities than building very short possessions and attacking by combinative attacks. No differences were observed in the likelihood of creating scoring opportunities depending on the contextual dimensions.

**TABLE 5:** Multilevel multinomial logistic regressing predicting to achieve scoring opportunity vs. penetration (Reference category).

**Dimension**	**Scoring opportunity vs. offensive penetration (univariate analysis)**			**Scoring opportunity vs. offensive penetration (multivariate analysis)**		
	**β**	**SE**	**OR (95% CI)**	**β**	**SE**	**OR (95% CI)**
**Initial penetration**						
No penetration (Ref)						
Penetration	0.636	0.167	1.889 (1.360–2.622)^∗∗∗^	0.312	0.197	1.367 (0.929–2.011)
**Initial pressure**						
Initial pressure (Ref)						
Non-initial pressure	–0.094	0.185	0.910 (0.633-1.309)			
**Duration of the attack**						
0–10 s (Ref)						
11–20 s	–0.128	0.188	0.880 (0.609–1272)	0.278	1.299	1.321 (0.868–2.011)
21–30 s	–0.651	0.262	0.521 (0.312–0.872)^∗^	0.272	0.788	1.313 (0.667–2.584)
31 + s	–2.296	0.273	0.744 (0.435–1.272)	0.866	0.229	2.376 (1.109–5.090)^∗^
**Type of attack**						
Combinative (Ref)						
Direct attack	0.996	0.448	0.369 (0.153–0.889)^∗^	–0.702	0.470	0.495 (0.197–1.245)
Fast attack	0.688	0.201	1.991 (1.342–2.952)^∗∗^	0.932	0.365	2.540 (1.430–4.513)^∗∗^
Counterattack	1.212	0.250	3.359 (2.056–5.488)^∗∗∗^	1.478	0.293	4.383 (2.144–8.961)^∗∗∗^
**Match location**						
Away (Ref)						
Home	0.057	0.179	1.058 (0.745–1.503)			
**Quality of opposition**						
Low-ranked (Ref)						
Medium-ranked	–1.149	0.215	0.862(0.565–1.314)			
High-ranked	–0.085	0.245	0.918 (0.568–1.484)			
**Match status**						
Losing (Ref)	0.063	0.225	1.065 (0.684–1.657)	0.087	0.242	1.091 (0.679–1.753)
Drawing winning	0.493	0.264	1.638 (0.976–2.747)^∗^	0.491	0.282	1.633 (0.940–2.839)
Losing (Ref)						
Drawing	0.063	0.225	1.065 (0.684–1.657)	0.087	0.242	1.091 (0.679–1.753)
Winning	0.493	0.264	1.638 (0.976–2.747)^∗^	0.491	0.282	1.633 (0.940–2.839)
Intercept				1.003	0.270	2.727 (1.605–4.632)^∗∗∗^

*β, Coefficient; SE, Standard error; OR, Odds Ratio; CI, Confidence interval for odds ratio; ^∗^*p* < 0.05, ^∗∗^*p* < 0.01, ^∗∗∗^*P* < 0.001.*

Table 6 shows the explanatory variables that predict the creation of goal scoring opportunities in comparison with all the rest of team possessions. Multivariate analysis showed how performing initial penetration, attacking with longer possessions and advancing by means of fast attack or counterattack registered higher probabilities in creating goal scoring opportunities than non-performing initial penetration, having very short possessions and attacking by means of combinative or direct attack, respectively.

**TABLE 6:** Multilevel binary logistic regression predicting to achieve scoring opportunity vs. no scoring opportunity (Reference Category).

**Dimension**	**Scoring opportunity vs. no scoring opportunity (Univariate Analysis)**			**Scoring opportunity vs. no scoring opportunity (Multivariate analysis)**		
	**β**	**SE**	**OR (95% CI)**	**β**	**SE**	**OR (95% CI)**
**Initial penetration**						
No penetration (Ref)						
Penetration	0.858	0.162	2.358 (1.717–3.237)^∗∗∗^	0.411	0.195	1.497 (1.022–2.192)^∗^
**Initial pressure**						
Initial Pressure (Ref)						
Non-Initial Pressure	0.290	0.181	1.337 (0.937–1.907)			
**Duration of the attack**						
0–10 s (Ref)						
11–20 s	0.405	0.180	1.500 (1.054–2.135)^∗^	0.724	0.209	2.084 (1.383–3.140)^∗∗∗^
21–30 s	–0.033	0.255	0.967 (0.587–1.594)	0.926	0.336	2.585 (1.337–4.998)^∗^
31 + s	0.397	0.265	1.488 (0.885–2.500)	1.642	0.379	5.057(2.406–10.627)^∗∗∗^
**Type of attack**						
Combinative (Ref)						
Direct attack	–1.493	0.442	0.225 (0.094–0.534)^∗∗^	–0.810	0.468	0.467 (0.187–1.169)
Fast attack	0.684	0.196	1.981 (1.350–2.908)^∗∗∗^	1.256	0.287	3.588 (2.045–6.294)^∗∗∗^
Counterattack	1.239	0.240	3.454 (2.156–5.533)^∗∗∗^	1.953	0.356	7.097 (3.530–14.269)^∗∗∗^
**Match location**						
Away (Ref)						
Home	0.227	0.172	1.255 (0.895–1.759)			
**Quality of opposition**						
Low-ranked (Ref)						
Medium-ranked	–0.145	0.205	0.865 (0.578–1.295)			
High-ranked	0.085	0.234	1.089 (0.688–1.725)			
**Match status**						
Losing (Ref)						
Drawing	–0.034	0.220	0.967 (0.629–1.487)			
Winning	0.302	0.254	1.353 (0.822–2.226)			
**Intercept**				–3.758	0.323	0.023 (0.012-0.044)^∗∗∗^
						

*β, Coefficient; SE, Standard error; OR, Odds Ratio; CI, Confidence interval for odds ratio; ^∗^*p* < 0.05, ^∗∗^*p* < 0.01, ^∗∗∗^*P* < 0.001.*

**FIGURE 4:**
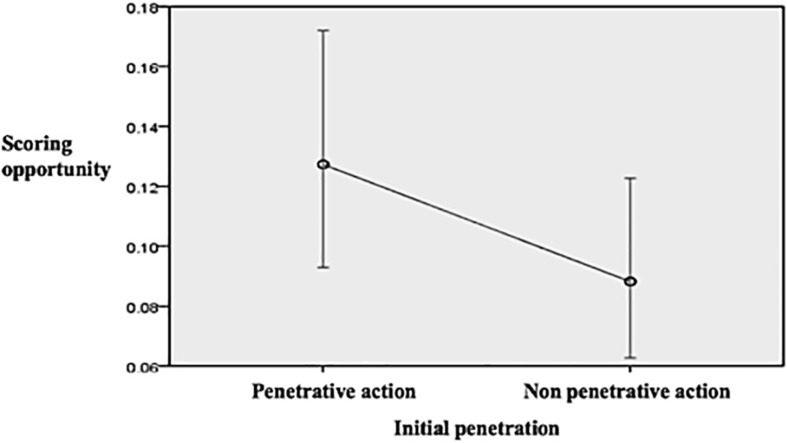
Predicted probabilities to create a scoring opportunity according to the level of initial penetration.

**FIGURE 5:**
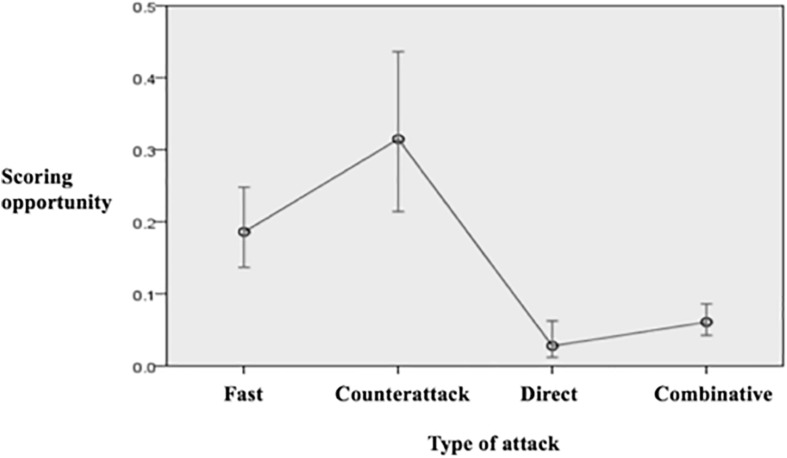
Predicted probabilities to create a scoring opportunity according to the type of attack.

**FIGURE 6:**
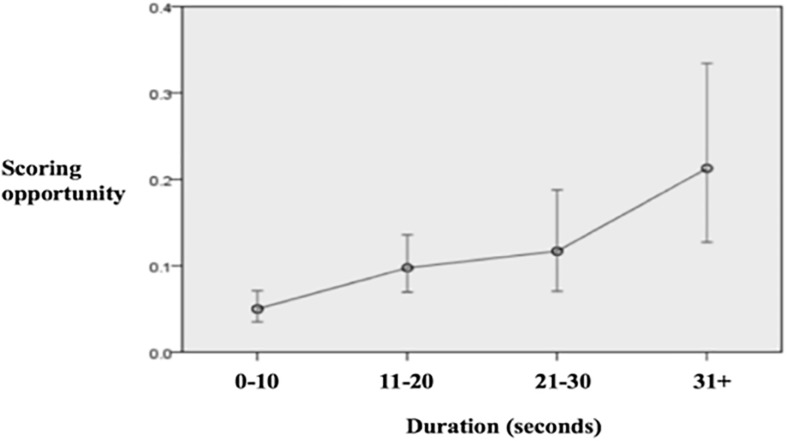
Predicted probabilities to create a scoring opportunity according to the duration of the attack.

In terms of creating goal scoring opportunities teams that started the possession with a penetrative action had a 13% of probabilities of producing a scoring opportunity while this probability was 9% for the teams that started the possession with non-penetrative actions (Figure 4). As for the type of attack, Figure 5 shows how nearly 1 out of 3 counterattacks and 1 out of 5 fast attacks produced a scoring opportunity, whereas the rate for the combinative and especially for the direct attacks was considerably lower. Figure 6 illustrates how as longer the team possession was sustained, the higher the rate of success was, highlighting those possessions that lasted 31 or more seconds that had more than 20% of possibilities of producing goal scoring opportunities.

## Discussion

The aim of this paper was to study the combined effects of tactical and contextual dimensions on achieving offensive performance in open play possessions from the Spanish “La Liga” soccer matches.

Firstly, our study explored the tactical characteristics of Spanish “La Liga.” This competition has been considered to have a more “possession-based” style of play in comparison with other European competitions (Sarmento et al., 2013; Mitrotasios et al., 2019). Our descriptive data supports this fact considering that nearly 75% of the team possessions that started in open play progressed by fast or combinative attacks, whereas the proportion of direct attacks and counterattacks was very low. In contrast, our results showed how the length of the team possessions was predominantly short so that only the 9.4% lasted more than 31 s. This fact may indicate the difficulty to sustain long ball possessions in the Spanish high-level soccer even if the main styles of play implemented by the teams are based on positional play. Also, our data revealed that 3 out of 4 team possessions started against defensive pressure and the majority of initial actions were not penetrative. These tactical characteristics reflect the high intensity context during the defense-attack transitions in this competition, where teams usually put pressure after losing the ball and create a highly demanding time-constrained scenario.

Secondly, our research looked for explanatory variables that can predict the offensive success. In regards to the start of the possession, our study found how performing initial penetrative actions was crucial to achieve both offensive penetration and scoring opportunities. Previous literature found similar results in different competitions. González-Ródenas et al. (2015) in Major League Soccer, Casal et al. (2015) in the Eurocup 2008 and Hughes and Lovell (2018) in the UEFA Champions League observed higher offensive effectiveness in team possessions that achieved to penetrate immediately after gaining the ball in open play situations. As for the initial defensive pressure, our data revealed how teams that play against no initial opponent pressure were more likely to achieve offensive penetration, although this fact did not increase the odds of creating goal scoring opportunities. In this way, our results support the idea that transitions periods in soccer present exciting opportunities and nervous vulnerabilities (Turner and Sayers, 2010) that are decisive to achieve tactical success, so that the offensive team has the chance of exploiting open spaces while the defensive team tries to reorganize their defensive system. For that reason, both the defensive and offensive tactical behaviors of teams during the transition moment may be decisive for the final outcome of the possession, contributing to the result of the matches and the overall performance of the team throughout the season, as previous studies have stated in different competitions (Vogelbein et al., 2014; Winter and Pfeiffer, 2016).

As for the development of the possession, univariate and multivariate analyses showed how fast attacks and specially counterattacks were more likely to achieve offensive penetration and scoring opportunities than combinative and direct attacks. In the same line, previous literature in different competitions found that counterattacks are less frequent but much more effective than combinative attacks (Tenga et al., 2010; Lago-Ballesteros et al., 2012; González-Ródenas et al., 2015; Sarmento et al., 2018). On the other hand, the study of Sarmento et al. (2018) found that fast attacks increased the success of an offensive sequence by 40% compared with combinative attacks in top European teams. These tactical findings suggest that both in a positional or a transitional scenario, building quick sequences with the intention of breaking opponent lines achieves higher degree of offensive success than an excessive passing combination or contrarily, excessive verticality by using long and direct balls.

As far as the possession length, longer possessions achieved higher degree of offensive penetration and scoring opportunities than very short possessions (10 s or less). The fact of building more elaborated possessions would contribute to have more opportunities to break opponent lines and get closer to the opposing goal. In this sense, there is still a debate about if shorter or longer possessions are more effective to achieve offensive success. Although a vast quantity of studies observed that longer possessions had more offensive effectiveness than shorter possessions (Hughes and Franks, 2005; Tenga et al., 2010; Lago-Ballesteros et al., 2012; González-Ródenas et al., 2015) other studies such as the one of Sarmento et al. (2018) reported that increasing the possession duration resulted in a decrease in the probability of success of the offensive sequence.

On the other hand, one of the strengths of our study is that our multinomial analyses also evaluated the effect of the tactical dimensions on creating goal scoring opportunities only in those possessions that achieved offensive penetration. In this tactical scenario, our analyses revealed how neither the initial penetration nor the initial opponent pressure presented differences in the odds of creating scoring opportunities. This may suggest that once the teams achieved to penetrate over the opposing lines, the origin of that penetration did not have a posterior influence on the final outcome of the possession, so other dimensions related to the development or the end of the team possessions would determine the creation of goal scoring opportunities. In fact, our study observed how fast attacks and counterattacks had greater odds of creating scoring opportunities than combinative attacks in penetrative possessions. These findings are interesting because confirm the greater offensive power of counterattacks and fast attacks in comparison with other types of attack even when only successful possessions were included in the analysis. This tactical effect may be due to the fact that counterattacks and fast attacks progress very quickly and this would not allow the opponent to readjust defensively. This situation would permit the offensive players to possess more time and space to disorder the last defensive line and create scoring opportunities. In contrast, successful possessions that progressed by means of combinative or direct attacks could allow the opponent to be closer to their defensive goal and have the possibility to reduce the space and time that the offensive players execute in order to create goal scoring opportunities.

Regarding contextual dimensions, our study observed how playing at home and playing against high-ranked opponents had greater effectiveness to achieve offensive penetration but no effect was found on the creation of goal scoring opportunities. This fact may be considered surprising so that multiple studies have found how contextual dimensions are related to several performance indicators and styles of play in soccer. In particular, home teams (González-Rodenas, 2013; Almeida et al., 2014), losing teams (Bradley et al., 2014; Fernandez-Navarro et al., 2018) and playing against weaker opponents (Castellano et al., 2013; Santos et al., 2017) have been associated with an increase in offensive indicators in different competitions. However, in the light of our results, more research is still needed to understand how different contextual dimensions may influence the creation of goal scoring opportunities.

As far as the limitations of our study, we are aware that our multidimensional analysis may not capture the high level of complexity that styles of play represent in soccer, where the constant interaction between teammates, opponents and contextual constraints create dynamic and interdependent situations that are different and unique in each match. We would recommend for future studies to evaluate the relationship between tactical dimensions and offensive performance by using other research methodologies such as spatial-temporal tracking data, (Memmert and Rein, 2018) temporal patterns (Camerino et al., 2012) or network analysis (Passos et al., 2011) that can offer a more complex approach in the understanding of offensive effectiveness. Secondly, our study only included the analysis of four tactical dimensions. In this regard, future studies should include more dimensions both offensive and defensive in order to obtain a broader number of performance indicators that characterize the styles of play and provide a better understanding of how offensive success is achieved in professional soccer. On the other hand, this study has important practical applications for coaches, sporting directors and tactical analysts so they can reflect on how offensive effectiveness is achieved in the Spanish “La Liga” and adapt their player development model, tactical strategy and recruitment process.

To conclude, the interactive effect of tactical dimensions such as initial penetration, type of attack and possession length played an important role on achieving offensive penetration and goal scoring opportunities in Spanish “La Liga” soccer matches.

## Data Availability

The datasets generated for this study are available on request to the corresponding author.

## Author Contributions

All authors contributed equally to the conception and design of the study, organization of the database, performing the statistical analysis, and writing the first draft of the manuscript. RA-M, FC, and RA, as corresponding authors, revised the final version. All authors approved the submitted version.

## Conflict of Interest Statement

The authors declare that the research was conducted in the absence of any commercial or financial relationships that could be construed as a potential conflict of interest.
